# A Case of Insulin Autoimmune Syndrome Accompanied With Systemic Joint Pain: A Case Report

**DOI:** 10.7759/cureus.47140

**Published:** 2023-10-16

**Authors:** Yuta Horinishi, Ryuichi Ohta, Chiaki Sano

**Affiliations:** 1 Community Care, Unnan City Hospital, Unnan, JPN; 2 Communiy Care, Unnan City Hospital, Unnan, JPN; 3 Community Medicine Management, Shimane University Faculty of Medicine, Izumo, JPN

**Keywords:** pancreatic cancer, ca 19-9, hypoglycemia, differential diagnosis, diagnosis, pancreatic enlargement, joint pain, spontaneous hypoglycemic episodes, autoantibodies, insulin autoimmune syndrome

## Abstract

Insulin autoimmune syndrome (IAS) is a rare disorder characterised by autoantibodies against endogenous insulin that cause spontaneous hypoglycemic episodes. Here, we present the case of a 66-year-old male with polyarticular pain and dizziness that was initially suspected to be an insulinoma. However, further testing confirmed the presence of IAS. The patient's joint pain fluctuated but improved with the control of blood glucose levels. Although the direct relationship between IAS and joint pain is not well established, individuals with a single autoimmune disorder may develop concurrent autoimmune conditions. Joint pain is prevalent in patients with autoimmune diseases. Although hypoglycemia may cause muscle cramps due to stress responses, direct musculoskeletal damage is uncommon. This case underscores the importance of differential diagnosis, particularly in differentiating between pancreatic cancer and the benign proliferation of pancreatic B cells. Elevated levels of insulin autoantibodies confirm IAS, whereas pancreatic cancer may manifest various symptoms and elevated cancer antigens (CA) 19-9. General physicians should comprehensively investigate hypoglycemia cases, particularly those associated with pancreatic enlargement, and continually monitor for potential malignancies.

## Introduction

Insulin autoimmune syndrome (IAS) is a rare condition in which patients develop autoantibodies against endogenous insulin without prior exposure to exogenous insulin [1]. This leads to episodes of spontaneous hypoglycemia due to autoantibodies binding to insulin, reducing its clearance, and simultaneously releasing large amounts of insulin [1]. In IAS, the immune system erroneously recognizes endogenous insulin or the insulin receptor as a foreign entity and generates autoantibodies against it [2]. These autoantibodies bind insulin and form insulin-autoantibody complexes [3]. Insulin activity is temporarily inhibited when bound to autoantibodies. Eventually, the insulin-autoantibody complexes dissociate, releasing a surge of active insulin into the bloodstream [1]. This sudden increase in bioavailable insulin can lead to hypoglycemic episodes. Rheumatologists may encounter IAS when evaluating patients with other autoimmune conditions, given the shared underlying mechanism of immune dysregulation [4]. The sudden onset of unexplained hypoglycemia in patients without diabetes or exposure to exogenous insulin may be a clue to this syndrome. Over time, many patients with IAS show spontaneous resolution of the condition; however, it may take months or years.

Insulin autoimmune syndrome is an autoimmune condition that can sometimes be accompanied by joint pain due to inflammatory processes, particularly when overlapping with other autoimmune conditions [5]. However, joint pain is not a hallmark of IAS [5]. Patients with a single autoimmune condition may be at higher risk of developing other autoimmune diseases, some of which may manifest as joint pain. Patients with one autoimmune disease may be at risk for other diseases. Conditions such as rheumatoid arthritis, lupus, and Sjögren's syndrome are potential culprits [1]. Moreover, some medications can induce joint pain; therefore, it is essential to consider the possibility of drug-induced arthralgia [6]. If a patient with IAS experiences joint pain, the patient must undergo a comprehensive evaluation by a rheumatologist or another appropriate specialist to determine the cause of the joint pain and receive proper treatment. Herein, we report the case of an older man with chronic systemic joint pain who was eventually diagnosed with IAS. In this case, the initial presentation was primarily musculoskeletal symptoms, which are rare in patients with IAS. Accordingly, we discuss the relationship between IAS autoimmunity and joint inflammation.

## Case presentation

A 66-year-old man visited a rural community hospital with a chief complaint of systemic joint pain and dizziness for two months. Two months before admission, the patient experienced systemic joint pain in the shoulders, elbows, wrists, and ankles with morning stiffness. The patient visited a primary care physician and was treated with acetaminophen (500 mg). One month before admission, the patient experienced dizziness between meals, which improved after consuming chocolate tablets. The pain and dizziness gradually worsened, and the patient revisited the primary care clinic on the day of admission. Laboratory tests revealed a blood glucose level of 45 mg/dL. The patient was then transferred to a community hospital for further investigation. The patient did not experience syncope, numbness, palpitations, chest pain, abdominal pain, back pain, cold sweats at night, or weight change. The past medical history included hypertension and brain stroke. The medications used were amlodipine (5 mg daily) and clopidogrel (50 mg daily) for 10 years. The patient did not take any herbal or over-the-counter drugs.

The patient’s vital signs at the visit were as follows: blood pressure, 132/86 mmHg; pulse rate, 67 beats/min; body temperature, 37.0 °C; respiratory rate, 16 breaths/min; and oxygen saturation, 97% on room air. The patient was alert to time, place, and person. Physical examination revealed tenderness in the bilateral shoulder, wrist, knee, and ankle joints without heat or swelling. No neurological abnormalities were observed. No abnormalities were observed in the chest, abdomen, or skin. Radiographs of the wrist and ankle revealed no bone abrasion. Laboratory tests revealed hypoglycemia and no inflammation (Table 1).

**Table 1:** Initial laboratory data of the patient eGFR: estimated glomerular filtration rate; Na: sodium; K: potassium; Ca: calcium; Cl: chlorine; P: phosphorous; Mg: magnesium; CK: creatine kinase; CRP: C-reactive protein; TSH: thyroid stimulating hormone; T4: thyroxine; Ig: immunoglobulin; Hbs: hepatitis B surface; HCV: hepatitis C virus; MPO-ANCA: myeloperoxidase-antineutrophil cytoplasmic antibodies; anti-SS-A/Ro antibody: anti–Sjögren's-syndrome-related antigen A autoantibodies; anti-CCP antibody: anti-cyclic citrullinated peptide (CCP) antibody

Parameter	Level	Reference range
White blood cells	7.2	3.5–9.1 × 10^3^/μL
Neutrophils	81.2	44.0–72.0%
Lymphocytes	8.0	18.0–59.0%
Monocytes	9.7	0.0–12.0%
Eosinophils	0.7	0.0–10.0%
Basophils	0.4	0.0–3.0%
Red blood cells	4.36	3.76–5.50 × 10^6^/μL
Hemoglobin	13.0	11.3–15.2 g/dL
Hematocrit	38.8	33.4–44.9%
Mean corpuscular volume	89.1	79.0–100.0 fl
Platelets	37.6	13.0–36.9 × 10^4^/μL
Total protein	7.9	6.5–8.3 g/dL
Albumin	3.5	3.8–5.3 g/dL
Total bilirubin	0.3	0.2–1.2 mg/dL
Aspartate aminotransferase	33	8–38 IU/L
Alanine aminotransferase	23	4–43 IU/L
Alkaline phosphatase	114	106–322 U/L
γ-Glutamyl transpeptidase	19	<48 IU/L
Lactate dehydrogenase	289	121–245 U/L
Blood urea nitrogen	11.6	8–20 mg/dL
Creatinine	0.54	0.40–1.10 mg/dL
eGFR	90	>60.0 mL/min/L
Serum Na	136	135–150 mEq/L
Serum K	3.4	3.5–5.3 mEq/L
Serum Cl	101	98–110 mEq/L
Serum Ca	8.9	8.8–10.2 mg/dL
Serum P	3.1	2.7–4.6 mg/dL
Serum Mg	2.4	1.8–2.3 mg/dL
CK	308	56–244 U/L
CRP	0.27	<0.30 mg/dL
TSH	2.3	0.35–4.94 μIU/mL
Free T4	1.2	0.70–1.48 ng/dL
IgG	1398	870–1700 mg/dL
IgM	42	35–220 mg/dL
IgA	494	110–410 mg/dL
HBs antigen	0.0	IU/mL
HBs antibody	0.00	mIU/mL
HCV antibody	0.00	S/CO
Syphilis treponema antibody	0.00	S/CO
SARS-CoV-2 antigen	Negative	Negative
anti-nuclear antibody	40	<40
MPO-ANCA	<1.0	<3.5 U/ml
anti-SS-A/Ro antibody	<1.0	<10.0 U/ml
anti-CCP antibody	<0.6	<5 U/ml
Urine test		
Leukocyte	Negative	Negative
Nitrite	Negative	Negative
Protein	Negative	Negative
Glucose	Negative	Negative
Urobilinogen	Negative	Negative
Bilirubin	Negative	Negative
Ketone	Negative	Negative
Blood	Negative	Negative
pH	7.0	
Specific gravity	1.013	

**Figure 1:**
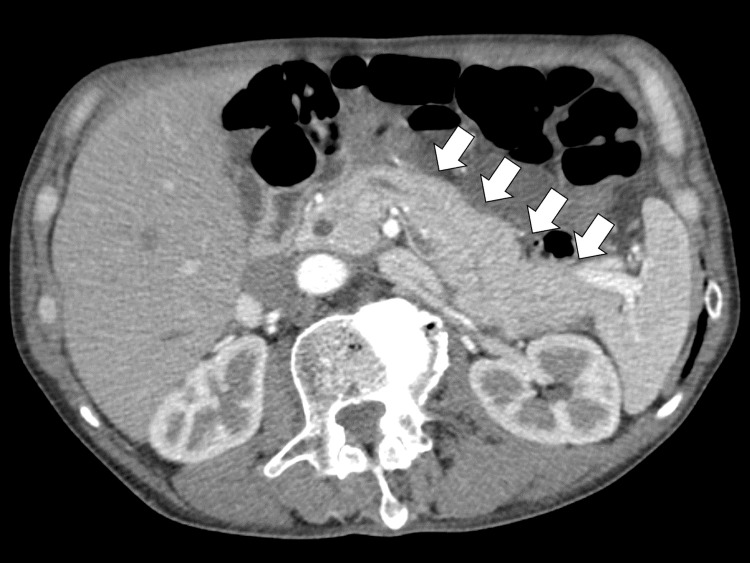
Enhanced abdominal computed tomography shows enlargement of the pancreas without masses in the pancreas and systemic lymphadenopathy (white arrows).

Initially, an insulinoma was suspected, and enhanced abdominal computed tomography (CT) revealed enlargement of the pancreas without masses or systemic lymphadenopathy (Figure 1).

Enhanced magnetic resonance imaging (MRI) revealed no pancreatic masses. Additional laboratory tests revealed a negative cancer antigen (CA) 19-9 of 10.4 U/mL (normal range: 0.0-37.0).

Based on previous findings, the patient was suspected of having insulin hypersecretion and the possibility of antibodies to insulin. The additional laboratory test showed a serum insulin level of 141.6 µU/ml (reference, <25 µU/ml), a C-peptide level of 2.3 ng/ml (reference, 0.5 to 2.0 ng/ml), an antibody-to-insulin level of ≧ 5,000 nU/ml, and an integration ratio of ≧ 90.0 % at fasting. Based on these findings, the patient was diagnosed with IAS. To prevent the surge of insulin secretion after eating, the patient was treated with an alpha-glucosidase inhibitor (αGI) of voglibose. The genetic investigation of IAS showed the aberration of DRB1*0407. Clopidogrel was considered for the possibility of triggering IAS, but the duration of the medication was long. Thus, it was assumed that the potential of the trigger could be low. The patient was trained to self-monitor glucose levels and was discharged with frequent hospital visits. During follow-up, the patient’s joint pain fluctuated but was alleviated with adequate control of serum glucose levels.

## Discussion

This case report shows that IAS can appear with the initial symptoms of joint and muscle pain and that pancreatic enlargement requires intensive investigation for the differential diagnosis between pancreatic cancer and monogamous proliferation of pancreatic B cells. During the gradual clinical course, general physicians should consider hypoglycemia a systemic symptom, such as joint and muscle pain and fatigue.

The relationship between IAS and joint pain, including the effects of hypoglycemia on the musculoskeletal organs, is complicated and intertwined. The patient’s joint pain fluctuated but improved after controlling for serum blood glucose levels. Insulin autoimmune syndrome is a rare cause of hypoglycemia characterized by spontaneous episodes of hypoglycemia and caused by autoantibodies against endogenous insulin [7]. These autoantibodies cause inappropriate and unregulated insulin release, leading to hypoglycemic episodes [1]. A direct correlation between IAS and joint pain has not been well established. However, individuals with autoimmune disorders often have multiple autoimmune conditions, and joint pain is a common symptom in various autoimmune diseases, such as rheumatoid arthritis and lupus erythematosus [8,9]. The direct cause and effect of the relationship between IAS and joint pain require further investigation. Hypoglycemia can also cause symptoms such as palpitations, tremors, sweating, and confusion with seizures [4]. Although not a primary symptom, muscle cramping or pain may occur during hypoglycemic episodes. This is generally due to the body's stress response and increased muscle activity, rather than directly affecting the musculoskeletal organs. Over time, severe and frequent hypoglycemia can have various detrimental effects on the body; however, specific musculoskeletal damage is not commonly reported [10]. This case report showed that controlling serum blood glucose levels can alleviate systemic joint pain; therefore, higher and lower glucose levels can affect joint pain. As system-specific specialists, general physicians should be concerned about serum glucose levels in patients with systemic symptoms.

Differentiating pancreatic cancer and the monogamous proliferation of pancreatic B cells in IAS is critical in general medicine. Our case highlights the importance of intensive investigations using multiple tests to rule out pancreatic cancer. Pancreatic cancer is a malignant tumor that grows in the pancreas and is one of the most lethal cancers worldwide. The symptoms include jaundice, unexplained weight loss, new-onset diabetes, and abdominal and back pain [11]. Diagnosis often involves imaging techniques such as CT, MRI, or endoscopic ultrasound. Tumor markers like CA 19-9 may be elevated [11]. The monoclonal proliferation of pancreatic B cells is related to IAS.

Moreover, IAS can be associated with the benign monoclonal proliferation of beta cells. This refers to the abnormal growth of similar pancreatic beta cells but is not a malignant-like cancer. Patients with IAS primarily present with hypoglycemia [5]. In contrast, the symptoms of pancreatic cancer vary more frequently and are often insidious. Pancreatic tumors or masses are observed in pancreatic cancer, whereas IAS may not necessarily show any masses on imaging, as shown in this case report. Elevated levels of insulin autoantibodies are a characteristic feature of IAS. Pancreatic cancer may show elevated CA 19-9, but this is not specific [12]. A biopsy can help differentiate between benign and malignant tissues if there is a pancreatic mass [13]. Thus, general physicians should investigate hypoglycemia with pancreatic swelling from multiple perspectives in collaboration with various professionals, including cases of the malignant and benign proliferation of beta cells in the pancreas, institute continual follow-up, and not wholly rule out pancreatic cancer [14,15].

## Conclusions

This case report highlights a rare IAS presentation characterized by autoantibodies against endogenous insulin, leading to spontaneous hypoglycemic episodes. In the present case, involving a 66-year-old male with systemic joint pain and dizziness, IAS was confirmed after an exhaustive investigation. Although a direct link between IAS and joint pain has not yet been established, this report underscores the importance of conducting thorough investigations into hypoglycemic cases, particularly when associated with pancreatic enlargement, and continuous monitoring for possible malignancies.
